# The societal opportunities and challenges of genome editing

**DOI:** 10.1186/s13059-015-0812-0

**Published:** 2015-11-05

**Authors:** Dana Carroll, R. Alta Charo

**Affiliations:** Department of Biochemistry, University of Utah School of Medicine, Salt Lake City, UT 84112-5650 USA; School of Law and Department of Medical History and Bioethics, School of Medicine and Public Health, University of Wisconsin, Madison, WI 53706 USA

**Keywords:** CRISPR-Cas, ethical issues, Food and Drug Administration, genetically engineered organism, germline genome editing, regulation

## Abstract

The genome editing platforms currently in use have revolutionized the field of genetics. At an accelerating rate, these tools are entering areas with direct impact on human well being. Here, we discuss applications in agriculture and in medicine, and examine some associated societal issues.

## Introduction

The genome editing technologies that are causing a current stir began life quietly in the 1990s, but are enjoying a remarkable surge, largely owing to the introduction of the CRISPR-Cas tools in 2012 [1–3]. The simplicity of that platform, compared with the earlier zinc-finger nucleases (ZFNs) and transcription activator-like effector nucleases (TALENs), has led to its rapid adoption and, in turn, to consideration of the uses to which it could readily be put. The power of these technologies derives from the fact that they allow directed modification of specific DNA sequences at their normal chromosomal locations, including changes as small as a single base pair or as dramatic as large deletions, insertions or translocations. The technologies have been used to produce models of human disease in experimental organisms and to explore fundamental gene function.

Current applications of genome editing include some with potential impact on the security of the world food supply and on clinical therapies. In fact, essentially the full range of uses — including agricultural and clinical, as well as potential nefarious ones — was already evident with earlier technologies, and many of the societal issues were recognized. The ethical issues surrounding human germline modification were partially addressed, even before the efficient nuclease-based technologies arrived. Here, we discuss briefly the capabilities of the genome editing technologies, their current and envisioned uses, and the relevant regulatory policies that are meant to reflect the public interest. Ultimately, the issues are whether the beneficial uses of genome editing are adequately safe and acceptable, whether regulatory oversight appropriately balances realistic risk assessment with achievement of the anticipated benefits, and whether there are any other factors that point towards promoting or impeding its use. In concert with several recent perspectives, we focus particularly on the potential for modification of the human germline.

## The technologies

It might not be widely appreciated that all the genome editing reagents accomplish is to make breaks in chromosomal DNA [4–6]. The ZFNs, TALENs and RNA-guided nucleases of the CRISPR-Cas system are fundamentally just nucleases. Their power comes from the fact that they can all be designed to make a break very specifically at essentially any target sequence that is chosen by the experimenter. This allows the modification of practically any locus in the genome of any organism.

**Fig. 1:**
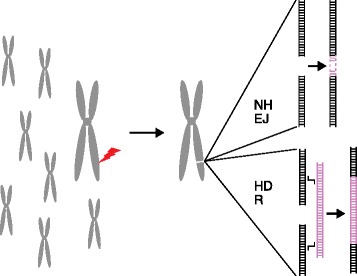
Pathways of repair after nuclease cleavage. In a cell with many chromosomes, a unique site on one chromosome is targeted for cleavage by a programmable nuclease (*red* ‘*lightning bolt*’). Cells repair the break either by non-homologous end joining (*NHEJ*), which can leave small insertions or deletions (indels), or homology-dependent repair (*HDR*) using a template supplied by the experimenter. Although mitotic chromosomes are illustrated here, it is unlikely that these processes occur specifically in mitosis

The modifications themselves depend entirely on the DNA repair capabilities of the cells in which the breaks are made [5]. In simple terms, essentially all cells and organisms rely on two broad types of process to repair double-strand breaks (Fig. 1). The ends at the break can simply be rejoined, either precisely or imprecisely, by a mechanism called nonhomologous end joining (NHEJ). Imprecise joining leaves behind small insertions or deletions (indels) at the break site, generating targeted mutations. When these are in coding sequences, they often constitute a knockout of gene function. Alternatively, repair can proceed by copying sequences from a template that has extensive homology with sequences around the break. This homology-dependent repair (HDR) would normally use a matched sequence on another cellular chromatid as a template, but it can be diverted to use a DNA supplied by the experimenter that carries desired sequence changes, leading to targeted sequence replacement.

**Fig. 2:**
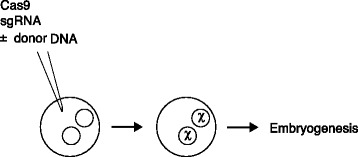
Illustration of one-cell embryo injection for CRISPR-Cas modification of a mammalian embryo. The nuclease components (the nuclease Cas9, and the short guide RNA (*sgRNA*)) are injected into a fertilized egg that has two nuclei derived from the male and female parents. The targeted modification (indicated by ‘*x*’) can occur in one or both nuclei, and the zygote proceeds to divide and form subsequent embryo stages

These repair mechanisms are common to a wide range of organisms, but, to benefit from their activity, the nuclease and template must be delivered effectively to the cells of interest. The delivery methods and the editing outcome will depend on the biology of the system. For example, the nucleases can be introduced into some organisms, including most mammals, by direct embryo injection, in conjunction with in vitro fertilization (Fig. 2). In most plants, however, delivery is more challenging (see section below on Genetically engineered organisms and their regulation). As a second illustration of the influence of the biological system, the balance between NHEJ and HDR varies considerably among cell types and organisms. HDR represents a significant proportion of events in rapidly dividing cells, but typically not in primary human cell cultures, and this limits the ability to make subtle intentional changes.

All of the nuclease platforms are capable of high specificity, but none of them is perfect. In the process of making desired changes at the designed target, unintentional changes can be induced elsewhere in the genome by cleavage and NHEJ repair at secondary sites [5]. This is a modest hazard in experimental organisms, where independent events can be compared, the genetic background can be cleaned up by out-breeding and conclusions can be validated by complementation with a wild-type sequence. There are also methods for detecting, locating and quantifying these off-target effects [7]. In applications to human therapy, we need to be assured that the treatment will not cause a new condition while curing the one intentionally addressed. Fortunately, the ability to direct subtle changes to the endogenous target avoids some of the dangers inherent in earlier methods for delivery of therapeutic genes (see below).

## Applications to agriculture

The current world food supply is inadequate, and the situation will get worse as populations continue to grow [8]. There are other serious considerations, including demands on uncertain water supplies, changing climates, and animal welfare. Genome editing will not provide general solutions to these broader issues, but there are some areas where the technology can help.

Applications to plants, including crops, are covered in detail below, but suffice it to say here that crops provide the bulk of nutrition for the world population. Any improvements in nutritional value and resilience would be welcome in many species, and some of these can be approached sensibly through genome editing [9].

In the realm of livestock, genome editing is just beginning to be applied, so specific applications are still emerging. One example that is being pursued currently is the genetic dehorning of dairy cattle [10]. Because the cattle are kept in close quarters, dairy farmers typically remove their horns by physical methods that are invasive, painful and expensive. Natural genetic variants, called polled, exist in some beef breeds [11]. This trait could, in principle, be transferred to dairy herds by traditional breeding, but it would be prohibitively time-consuming and expensive to do so as it would be necessary to perform extensive additional breeding to restore favorable dairy traits. Because the responsible DNA sequence change has been characterized, it is possible to use genome editing [12] to introduce the variant into existing herds without affecting their other, beneficial, traits. The result would be the addition of the polled allele to the dairy genomes, with no additional DNA being present.

Another application envisioned for cattle and for pigs is mutation of the myostatin gene, which negatively regulates the production of skeletal muscle. Natural mutations in this gene exist [13]. Homozygous mutants are rather grotesquely muscled, but heterozygotes are largely normal, except that they have approximately 7 % more muscle mass in the form of lean, marketable meat. Such mutations can readily be produced in cells [14, 15], and a recent news report indicates that live pigs have been generated carrying myostatin mutations [16]. These genetic maneuvers can be performed independently in breeds that carry adaptions to different environmental conditions, such as heat or cold tolerance, drought tolerance, or resistance to particular infectious agents. In addition, as the genetic variants responsible for those adaptations are identified, they could also be introduced into new breeds by genome editing.

## Genetically engineered organisms and their regulation

Quite literally, genome-edited animals and plants are genetically modified organisms — GMOs — but they differ from the controversial genetically engineered crops currently under cultivation. The latter carry transgenes imported from other species, commonly from bacteria. By contrast, genome editing allows the precise inactivation of an endogenous gene, the conversion of an existing allele to a more favorable one, or the precise insertion of an identified variant into additional breeds. The animal and plant products of these modifications are essentially identical to ones that could, and in some cases do, occur naturally or could be created by traditional breeding methods. Because editing is performed in a hit-and-run fashion — the nucleases do their job and then are degraded within cells — no trace of the reagents remains in the organism. For considerations of safety, it seems sensible to regulate based on the product's characteristics, independent of the process used to develop them.

In the USA, genetically engineered plants are subject to regulation by three federal agencies: The United States Department of Agriculture (USDA) Animal and Plant Health Inspection Service, the Department of Health and Human Services' Food and Drug Administration (FDA), and the United States Environmental Protection Agency (EPA). If a major federal action results, there might also be a requirement for public review and consultation under the National Environmental Policy Act [17].

Since the development in the 1980s of the ‘coordinating framework’, it has been US policy to regulate biotechnology products based on their characteristics and intended uses, and not by their method of production, even when that method involves novel technologies. The approach has been mirrored in other areas as well. Nanotechnology, for example, is the subject of a great deal of discussion among the many departments for which it is relevant, ranging from workplace protections to environmental safety to evaluation of new drugs, devices and foods, but in the end, each nanotechnology product is regulated according to the product’s standard pathway.

As a general rule, products are regulated under existing law, and the method of production is relevant only to the extent that it affects the considerations required under existing law. For example, the USDA will look to see whether a new kind of plant constitutes a ‘plant pest’, and will examine the extent to which the engineering changes characteristics of the plant, which will be examined to see whether the organism now grows, spreads or competes in ways that would make any other plant a ‘pest’. The EPA looks at the safety of pesticides, and will similarly look at the safety of ‘plant-incorporated protectants’ produced through genetic engineering. For the FDA, reviewing the safety of a human or animal drug includes looking at long-term effects, including the stability or off-target effects of any genetic changes. And if a vector (regarded as an animal drug) is used for a food animal, the product will be reviewed for safety in the animal, the environment and the resulting food.

To some extent, this differs from European approaches [17–19], where the use of genetic engineering — regardless of the resulting characteristics of the product — will trigger special requirements, for example, product labeling. In general, there is greater pre-market control, whether for deliberate release of organisms or sale for food and animal feed, based on a more aggressive interpretation of the precautionary principle and fewer limitations on government authority to prohibit or compel commercial speech. The situation is complicated by the division of authority between the governmental bodies of the European Union and those of individual member states, and recent debates have focused on the degree of autonomy that should be allowed at the national level.

The researchers and companies, in the USA and elsewhere, who are dedicated to genome editing of crops and livestock certainly hope that the simplicity, precision and naturalness of the modifications will lead to public acceptance of the products. Much of the opposition to genetically engineered organisms, however, is political, economic and visceral, and the scientific distinctions might not carry much weight. Economic concerns encompass distrust of corporate agriculture, resistance to awarding intellectual property rights for seeds, and fear of disrupting local industries dependent on wild-caught or heirloom varieties of animals and plants. They also encompass fear of unintended ecological consequences. And beyond this, for many people there is an emotional attachment to a particular conception of nature and of genetics, one that might not conform to biological definitions, but which is part of a world view in which man-made modifications and products using modern genetics are seen in part as evidence of hubris. Therefore, while genetically modified crops are demonstrably safe to eat, both by livestock and people, it might be difficult to overcome a fundamental resistance to intentional genetic manipulation, despite the fact that selective breeding by humans has produced the genomes of essentially all the foods we currently consume.

## Applications to medicine

Ever since the discovery of specific human disease genes, scientists have harbored hopes that the responsible mutations could be reversed with molecular approaches. In cases where a gene product is frankly missing, it is possible to provide a functional copy of the gene. While this has been successful in a few cases, several challenges stand in the way of facile use of the approach, including delivery of the gene to the affected cells, safe and efficient integration into the genome, and immunological reaction to the therapeutic protein itself or the vector used for delivery. Two early clinical trials dramatically illustrate the hazards. In tests of gene therapy for ornithine transcarbamylase deficiency, a teenager died as a result of a severe immune reaction to the adenoviral vector [20] after receiving a very high dose of the therapeutic vector in an attempt to deliver a curative level of gene expression.

A program to treat cases of X-linked severe combined immunodeficiency (SCID-X1) relied on an ex vivo approach, delivering the missing IL-2Rγ gene to bone marrow stem cells from the patients, followed by re-implantation. This led to sustained reversal of the immune deficiency in a majority of the recipients [21]. In several cases, however, leukemias developed owing to activation of the *LMO2* oncogene caused by integration of the retroviral vector in its vicinity [22]. The frequency of the offending integrations was very low in the treated bone marrow cells, but they had a significant growth advantage once expanded and returned to the patient’s body. This experience put a damper on therapies using retroviral vectors.

More recently and for the first time, a gene therapy procedure has been approved in Europe for treatment of patients. Glybera delivers the gene encoding lipoprotein lipase in an adeno-associated virus (AAV) vector to muscle tissue in patients lacking this enzyme [23]. AAV has the advantage that it does not integrate into the human genome, or does so rarely at an innocuous site, so it is judged safer than retroviral vectors.

Genome editing has several advantages relative to vector-mediated gene delivery: (i) in most cases, a mutant gene will be corrected, or otherwise modified, at its normal genomic locus, so random integration is not an issue; (ii) because editing occurs at the normal locus, no sequences driving expression need to be present on any donor DNA, and regulation of the target gene will be normal; (iii) off-target mutagenesis, if it occurs, is unlikely to activate a gene, as was seen in the SCID-X1 trials; and (iv) the editing materials will be present only transiently in the cells, and only the edit itself will persist.

One example of genome editing applied to the clinic has already been published [24]. It was based on the observation that some humans naturally lack a functional gene for CCR5, the required co-receptor in T cells for most strains of HIV-1. Although these people can be infected with the virus, the immune system is not depleted because their T cells are resistant to killing. A pair of ZFNs that was very highly evolved for efficacy and specificity was used to treat T cells derived from HIV-infected patients during laboratory culture. The *CCR5* gene was mutated in a substantial fraction of the cells, including some biallelic knockouts. The treated cells were returned to the patient from whom they were derived. Although therapeutic value was not the goal of this phase I trial, the procedure showed no ill effects, and the mutated cells persisted for a remarkable period of time. An extension of this approach will likely apply the same treatment to bone-marrow-derived hematopoietic stem cells (HSCs), in which successful knockout of CCR5 would provide a long-term reconstitution of an HIV-resistant T cell arm of the immune system.

Like this trial and the one for SCID-X1, the future therapeutic applications that are easiest to envision are ones involving ex vivo treatment. The treated cells can be analyzed in vitro to ensure that the desired modification has been made, and successfully modified cells could potentially be enriched, before implantation in the patient. If, and when, therapies based on stem cells other than HSCs are developed, genome editing and autologous transplantation will be useful there as well. Direct delivery to tissues in the body, by contrast, presents serious challenges. Consider, for example, the case of cystic fibrosis, where multiple tissues are affected, and success would require delivery to epithelial cells deep in the lung.

## Human germline modification

We must start this section by pointing out that discussions of the scientific and ethical considerations surrounding genetic modification of the human germline were initiated long before current technologies were envisioned (e.g., see [25]). The apparent facility with which such modifications can now be accomplished has made discussion of the issues more urgent. Historically, essentially all participants in these discussions have called for broad consideration of the issues by representatives of many perspectives: scientific, philosophical and societal. This call was reiterated very recently by members of both industry and research communities [26, 27], and has become the subject of a joint initiative by the National Academy of Sciences and the National Academy of Medicine that will focus equally on domestic and transnational possibilities and concerns [28].

The methods for performing germline editing on nuclear DNA are already available. They have been applied to other mammals, including primates [29–31], and one account of their use in non-viable human embryos has been published [32]. To achieve a transgenerational modification of the germline, two approaches are possible, both performed in conjunction with in vitro fertilization and then gestation of the resulting embryo. One is to make the desired modifications in cultured cells and transplant a nucleus from a successfully modified cell into an enucleated egg fertilized in vitro. This is called somatic cell nuclear transfer (SCNT) and is sometimes referred to as ‘cloning’. This approach can be readily dismissed, at least for current purposes, because experience with several different animal species has shown that it is associated with a very high frequency of developmental defects, presumably owing to the difficulty of reprogramming a somatic cell nucleus for all developmental functions.

The second approach is to deliver the editing materials (nuclease with or without donor DNA) directly to a fertilized egg and let the modifications take place there in the maternal and paternal genomes (Fig. 2). Implantation of eggs fertilized in vitro shows a high success rate that would probably not be much affected by the editing procedure itself. With injection, there is a significant chance that the embryos will be mosaic for the modification, if some nuclease cutting occurs after cell division and the efficiency is less than 100 %. There is also the danger of off-target mutagenesis, and it will be challenging to assess this at a sufficiently early stage.

In the long run, germline editing might proceed by modifying gametes before fertilization. This will require not only effective methods for delivering the reagents, but an understanding of the DNA repair capabilities of sperm and eggs.

## Ethical considerations

For the moment, despite the plethora of other possible applications, much of the most impassioned discussion about CRISPR-Cas9 has focused on its potential for editing the nuclear DNA of human gametes or embryos — so-called germline editing. The critiques largely break down into two large categories that are used in ethical analyses of many different kinds of technologies and human actions. The first — which is present in some religious analyses, but is also the hallmark of secular approaches — might be called consequentialist [33]. On the one hand, it focuses on the possibilities for improving the human condition, through the elimination of deleterious characteristics or mutations. It might allow people who carry such traits to have children to whom they are genetically related without the prospect of passing on problematic or dangerous conditions. To the extent these changes would persist across the generations, it could benefit not only the immediate offspring, but also all of the descendants of those who use the technology. On the other hand, it is this same phenomenon — of a change that reverberates down through the generations — that increases concern about unintended effects whose disadvantages might grossly outweigh any advantages that genome editing confers. And, because these risks would be borne by those who had no say in the decision, it eliminates the most common justification for such actions — that is, that the risk-taker has made an informed and voluntary decision to encounter the risk. While this is certainly true in every case of parental decision-making on behalf of a future or existing child, in those situations the rearing parents will share with the child both the risks and the possible benefits, thus adding some situational constraints on rash action. But when those risks and possible benefits are largely felt by future generations, this constraint, in the form of self-interest and self-protection, is removed.

Critics will also point to the intrinsic uncertainty about downstream effects, and will invoke some form of the precautionary principle [34], which demands a strong justification before permitting any risk-creating activity, with risk defined both in terms of known hazards and unknown possibilities. The latter, of course, is incapable of measurement, which is where the precautionary principle can be stretched into a generalized prohibition. In cases of devastating genetic diseases, some might argue forcefully that the risks of editing procedures are acceptable. At the same time, we must admit that we cannot confidently predict all the consequences, whether of introducing deleterious traits or by losing unanticipated benefits to retaining particular alleles. The heterozygote advantage of the sickle cell hemoglobin mutation in resisting malaria infection comes to mind.

As to the justification for taking risks, a variety of means already exist to avoid passing on problematic traits, including the choice to forego biological reproduction, the use of donated gametes and embryos, or the use of pre-implantation and prenatal diagnostic techniques to avoid the birth of an affected child. Even while acknowledging that the option of embryo selection or selective abortion will be unacceptable or emotionally difficult for many, the availability of these alternatives will be seen as a means to diminish the prospective benefits of gene editing, by measuring those benefits solely in terms of marginal increases in personal choices and good birth outcomes.

Another thread in consequentialist argumentation concerns the wisdom of any effort to alter the human condition through genetic manipulation. Even before the glimmerings of a theory of genetic inheritance, societies across the world had eras in which they viewed selective breeding as a means to ensure the superiority of any resulting children. With the publication of Darwin’s works, and their manipulation into social theory by Herbert Spencer, a new age of ‘scientific’ eugenics was born. Couched in terms of social hygiene, it attracted followers from all parts of the political spectrum and combined crude understandings of genetics with a host of cultural prejudices. Not surprisingly, it led to ugly decades of the worst form of eugenics, with mass involuntary sterilizations and mass murder [35]. Genome editing, like its less efficient predecessors (including choice of gamete donors, or pre-implantation selection of embryos), is touted by some for its potential to clear deleterious traits from the family line, and criticized by others for its echoes of simplistic and cruel notions of genetic superiority and inferiority [36].

Closely connected to these concerns, but with some independent factors, is a second standard form of ethics analysis, one that focuses less on specific consequences and more on some set of fundamental principles of right and wrong, or on spiritual and religious views about the appropriate scope of human control over the planet and the species. These categorical approaches are frequently found in theological analyses of new biotechnologies. For example, towards the end of Simon Mawer's 1998 novel, *Mendel's Dwarf* [37], the protagonist, a hereditary dwarf, faces a choice:

*"Benedict Lambert is sitting in his laboratory playing God. He has eight embryos in eight little tubes. Four of the embryos are proto-Benedicts, proto-dwarfs; the other four are, for want of a better word, normal. How should he choose?"*

For those approaching the question from a religious point of view, many see the act of choosing as a usurpation of God’s role in mankind’s existence. During a 1997 consideration of cloning policy, for example, the National Bioethics Advisory Commission (NBAC) [38] listened as theologian Dr Gilbert Meilaender testified that Protestants, although stout defenders of human freedom, nonetheless "have not located the dignity of human beings in a self-modifying freedom that knows no limit, [not] even…God." Rev. Albert Moraczewski, a Catholic, testified that cloning "exceed[s] the…delegated dominion given to the human race. There is no evidence that humans were given the power [by God] to alter their nature or the manner in which they come into existence" [38]. But in the novel [37], Benedict's instinct about God's role is somewhat different:

*“Of course we all know that God has opted for the easy way out. He has decided on chance.... You may…select two of the four normal embryos and send them over to the clinic for implantation …or…select the four achondroplastics, the four stunted little beings…and send them over instead…or… refuse to usurp the powers of God and choose instead to become as helpless as He…by choosing one normal embryo and one achondroplastic and leaving the result to blind and careless chance.”*

It is evident that Americans do not share a common view on the act of choice where creating and altering life is concerned. While some see choosing as ‘playing God’, others see it as ‘playing human’. Indeed, Rabbi Elliot Dorff testified at that same NBAC meeting that we are "the partner of God in the ongoing act of creation. We are God's agent.... ". Examining Biblical texts, Rabbi Moshe Tendler testified that being such a partner means taking an active role, and that ‘artificiality’, far from being wrong or evil, is rather a sign of humanity's constructive contribution, a sign that we are doing our duty. Furthermore, a professor of Islamic studies, Aziz Sachedina, described how the Koran suggests that "as participants in the act of creating with God, God being the best of creators, human beings can actively engage in furthering the overall state of humanity by intervening in the works of nature, including the early stages of embryonic development" when the goal is to achieve a natural good, such as health or fertility [38].

It is equally evident that people around the globe do not share a common view on the act of choice where creating and altering life is concerned. In places such as Singapore, China or Israel, attitudes about the moral and legal significance of embryos and fetuses, and about the appropriate degree of human control over its environment and its destiny, have been shaped by different histories and religious traditions [39]. In Germany, where the events of World War II still loom large in the collective memory, anything that relates to genetics will be met by skepticism, especially if there is any hint of eugenics [40]. In France, the internal politics of a country dedicated to secularism since the 18^th^ century but with powerful church influences has led to a degree of conservatism with respect to all forms of embryo research, and will likely have the same effect on debates about whether to make changes in the human germline [41]. By contrast, the United Kingdom has spent decades building a regulatory apparatus that is integrated with public opinion and legislative oversight, and which is allowed by law to exercise tight control not only over technologies, but even over every particular use of a technology, down to the laboratory, clinic and patient, something not often possible under the US system [42]. It should be no surprise, then, that the use of gene editing will likely proceed at wildly different rates among countries, cultures and regulatory systems.

To address this reality, a small group of scientists, lawyers and ethicists came together in early 2015 in Napa, California. The discussion there led to a call for a temporary moratorium on human applications of germ-line editing [26]. This was quickly followed by an announcement by the National Academy of Sciences and National Academy of Medicine that a joint initiative would be undertaken, with two major activities [28]. The first, an international summit, would gather scientists and thought leaders from around the globe, to discuss the state of the research around the world, to compare regulatory and cultural approaches, and to begin thinking about the kind of global norms that might be most appropriate to this area. The second, a study committee, will dig more deeply into the science, with an eye to understanding probable applications, their risks and benefits, and the applicable oversight systems.

In advance of these deliberations, The Hinxton Group [43], a self-organized international group of scientists and ethicists, has recently issued a statement on genome editing technologies and human germline modifications. Like others who have entered this discussion, they believe that technical advances are necessary before human germline applications should be undertaken. At the same time, they appear to make a tacit assumption that such manipulations will ultimately go forward, and, in this context, recommend that research on genome editing in human embryos should proceed under strict guidelines. While acknowledging the ethical concerns, they caution against over-regulation, which could inhibit orderly progression towards legitimate uses of the technology.

Even further along this path is the UK’s Human Embryology and Fertilisation Authority, which is now considering a specific proposal for use of gene editing on human embryos, in order to investigate the causes for repeated miscarriages [44]. The British and American systems of governance are quite different. In the USA, this procedure would likely be under the jurisdiction of the FDA, which would evaluate preclinical and clinical research data for a particular indication. If approved, the procedure could be advertised and promoted for only that indication and patient population, but physicians would have discretion to use it for indications or types of patients other than those for which it was approved. By contrast, in the UK, control over use is tighter — physicians and clinics must be licensed for each application. This allows for more precise control over dissemination of the technique, but at the cost of losing a degree of professional independence and judgment.

## Concluding thoughts

Genome editing, whether with ZFNs or TALENs or, now, with CRISPR-Cas (see also Box 1), represents a next step in our ability to analyze and alter the genetics of plants and animals, including ourselves. The notion that knowledge and the choices it offers might be our downfall is as old as the biblical tale of the Garden of Eden. But, in equal measure, history demonstrates the enormous benefits in health and happiness that come with responsible exercise of our intellect and powers of invention. The newest developments in genome editing will demand that we think again about how to balance hope and fear.

## Box 1. Gene drives

An additional use of genome editing, particularly of the CRISPR-Cas tools, is envisioned in applications called synthetic ‘gene drives’ [45]. As a general term, gene drive refers to DNA sequences — sometimes whole chromosome sets — that increase the frequency of their own inheritance. There are several natural examples, but the current discussion focuses on the construction of such elements for the control of populations of disease vectors [46] — for example, the tropical mosquitoes that spread the malaria and dengue fever agents. The approach would be, for example, to introduce into the *Anopheles* genome a nuclease gene that cuts a crucial target — perhaps a gene required for *Plasmodium* transmission. Upon cleavage, copying of the nuclease’s own coding sequence into the target site is stimulated. Not only would this inactivate the target gene, the inherited allele would in turn induce copying of the insertion into a vacant allele in the next generation. Thus, the mutation would spread rapidly through the breeding population.

There is appropriate concern that spread of the gene drive will be difficult to control, and it might spread to populations or have consequences beyond those intended [47]. Various designs of the drive itself and other containment measures have been proposed to prevent such escapes. While the gene drive scheme could, in principle, be executed with any of the nuclease platforms, it is again the efficacy and simplicity of CRISPR-Cas that has suggested that such applications are close at hand [48] and deserve careful examination.

## Abbreviations

AAV: adeno-associated virus
Cas: CRISPR-associated protein
CRISPR: clustered regularly interspaced short palindromic repeats
EPA: Environmental Protection Agency
EU: European Union
FDA: Food and Drug Administration
GM: genetically modified
GMO: genetically modified organism
HDR: homology-dependent repair
HSC: hematopoietic stem cell
indel: insertion or deletion
NHEJ: non-homologous end joining
SCID-X1: X-linked severe combined immunodeficiency
SCNT: somatic cell nuclear transfer
TALEN: transcription activator-like effector nuclease
USDA: US Department of Agriculture
ZFN: zinc finger nuclease
